# Editorial: Vesicular Trafficking in Cell Communication: New Insights in Physiology and Pathology

**DOI:** 10.3389/fcell.2021.772306

**Published:** 2021-10-25

**Authors:** Anna Onnis, Alvaro H. Crevenna

**Affiliations:** ^1^Department of Life Sciences, University of Siena, Siena, Italy; ^2^Epigenetics and Neurobiology Unit, European Molecular Biology Laboratory Rome, Rome, Italy

**Keywords:** cell communication, vesicular trafficking, extracellular vesicles, endosomes, lysosomes

Cells of multicellular organisms communicate with each other to maintain homeostasis or to adapt when in presence of stress signals. Communication may occur through one or more of several ways: direct cell-cell contact, where gap junctions or adherent proteins facilitate signal propagations, cell-matrix interaction by integrins which allow long-range signals such as hormones that are released in the blood stream as well as proteins (SDF-1, HGF, and FGF/RA), nucleotides, lipids, and short peptides to monitor the close environment (Lodish et al., 2000). In addition, the release of vesicles into the extracellular space, called extracellular vesicles (EVs), which contain a cell-specific cargo of proteins, lipids, and genetic material, adds a further layer of complexity in this key biological process (van Niel et al., 2018). Within the cell, vesicles continually bud off from one membrane and fuse with another, carrying membrane components and soluble molecules referred to as *cargo*. This vesicular trafficking flows along highly organized, balanced and directional routes from the ER toward the Golgi apparatus and cell surface or toward lysosomes (the biosynthetic-secretory pathway) and from the plasma membrane to early endosomes and recycle them back to the surface to be reused or sort them to degradation (the endocytic pathway; McMahon and Boucrot, 2011). Disorders in this finely organized vesicular trafficking in and out of the cells is a hallmark of cancer and other disease states.

In this Research Topic we have collated original research and review articles from many different subtopics on cellular communication in pathophysiological processes in order to cover the various relevant aspects of this interesting topic.

In a multi-omics study of cell-to-cell communication, Royo et al. described the effects of the release of EVs derived from liver progenitor cells in fibroblasts. Transcriptomic and proteomic analysis revealed a decrease in pathways related to catabolic activity, cell proliferation, cytoskeleton remodeling, and carbon metabolism. In parallel, EVs-treated fibroblasts showed an increase of signals related with immune system activation and ribosome biogenesis, suggesting overall a tissue damage response. These results reinforce the ability of EVs to initiate regenerative processes in recipient cells. Of note, EVs are released not only from progenitor cells but also from differentiated cells in liver. In an elegant study, Li et al. reported for the first time that the adesive glycoprotein fibronectin (FN1) is a constituent of hepatocyte-generated EVs. Using FN1 knock-out cells, the authors characterize the role of FN1 hepatocyte-EVs. FN1 facilitates the integrin-dependent EV binding and EV uptake by endocytosis of target cells such as hepatocytes and hepatic stellate cells (HSC) but is dispensable for EV-mediated anti-fibrotic activity *in vivo*. A fascinating role in the use of EVs in the improvement of therapy strategy for the type 1 dyabete mellitus (T1DM) is highlighted by the work of Bai et al. The authors improved a previously characterized protocol for beta cell generation from iPSCs using miR-212/132-enriched EVs secreted by beta cells. Functional analysis defined the role of the miRNAs 212 and 132 in the regulation of target mRNAs important to promote differentiation of endocrine cells from induced iPSCs. Importantly, the generated beta cells from miR-212/132-enriched EVs are also able to secrete insulin under glucose stimulation and ameliorated hyperglycemia *in vivo*. All together these original works support the importance of the application of EVs both in physiology and pathological contexts due to their pivotal roles in cross-talk intercellular communication. Accordingly, Bergamelli et al. investigated the expression of surface markers of placental EVs after optimization of their isolation from a histoculture model of first trimester placental explants in normal conditions and after human cytomegalovirus (hCMV) infection. The authors found that a number of molecules changed in the surface of EVs after infection. This paper paves the way for downstream functional characterization of these non-invasive biomarkers that could be relevant for the outcome of the viral infection during pregnancy.

The main form of intracellular communication occurs through the trafficking of vesicles between several compartments, as exemplified by the endosomal-lysosomal system. Interestingly, the latter intersects the autophagic system thus increasing the complexity of the cross-talks between these cellular processes. Unraveling these pathways, the original article by Finetti et al. brings to light to the identification of the intraflagellar transport protein IFT20 as a new regulator of the autophagic system. In particular, the authors found that IFT20 plays a direct role in autophagy as an adaptor that targets ATG16L1 and downstream autophagy regulators to a specific endomembrane compartment, resulting in autophagosome biogenesis. This discovery confers a new role for IFT20 in T cells beyond its implication in lysosome biogenesis, as recently demonstrated from the same group. In the original article submitted by Wen et al., another new player well-known for vesicular transport, the Golgi brefeldin A resistant guanine nucleotide exchange factor 1 (GBF1) protein, was also identified to play a role in autophagy in bone-resorbing osteoclasts (OCs). In particular, the authors show that the GBF1 inhibition impairs not only *in vitro* differentiation of osteoclasts but also causes ER stress and autophagy through alterations of different signaling molecules. Bodakuntla et al. summarize the crucial role of two important structural components of the vesicular trafficking (cytoskeleton and membranes) in axonal branches in which the local machinery dynamically controls and regulates the axonal growth to ensure a high degree of interconnectivity for neural network. The dysfunction of the endosomal-lysosomal system is nicely illustrated in the context of atherosclerosis, a disease whose etiology and pathogenesis are strictly dependent from defective lysosome functions. The review of Marques et al. fully describes the origin and impact of the malfunctioning of lysosomes in plaque cells in cardiovascular diseases. Moreover, they also reviewed the field of Lysosomal Storage Diseases (LSDs) research to point up how therapeutic approaches that target lysosomes in LSDs might hamper atherosclerosis progression and its high associated mortality.

Notwithstanding the continuous growth within the field of vesicles in cell communication, many unanswered questions remain that hold still several future perspectives. For example, what are the biological implications and functions of the extra- and intracellular vesicles size diversity? How can we generate a more comprehensive understanding of the molecular machinery and events of protein trafficking? Does the variety of vesicles produced or released by a defined cell type change over time? How do vesicles are spatiotemporally coordinated? How can modulate vesicle targeting to improve therapeutic strategies? These are important future challenges that we hope will bring light to our understanding of new functional and structural properties of cell communication through vesicles with a significant impact not only on translational medicine but also on more general fields, such as cell biology, virology and immunology.

We would like to thank the authors and reviewers for their substantial contribution in participating on this Research Topic. Altogether, these studies significantly improve our knowledge on cellular communication and we hope they could encourage the birth of future works in this field to provide an ever better understanding on how the communication in-and-out of the cells occurs and how its dysregulation may be affected in disease.

## Author Contributions

AO conceived and wrote the Editorial. AC reviewed and edited the manuscript. All authors contributed to the article and approved the submitted version.

## Conflict of Interest

The authors declare that the research was conducted in the absence of any commercial or financial relationships that could be construed as a potential conflict of interest.

## Publisher's Note

All claims expressed in this article are solely those of the authors and do not necessarily represent those of their affiliated organizations, or those of the publisher, the editors and the reviewers. Any product that may be evaluated in this article, or claim that may be made by its manufacturer, is not guaranteed or endorsed by the publisher.
